# Astaxanthin *n*-Octanoic Acid Diester Ameliorates Insulin Resistance and Modulates Gut Microbiota in High-Fat and High-Sucrose Diet-Fed Mice

**DOI:** 10.3390/ijms21062149

**Published:** 2020-03-20

**Authors:** Yuan Gao, Lu Yang, Yaoxian Chin, Fang Liu, Robert W. Li, Shihan Yuan, Changhu Xue, Jie Xu, Qingjuan Tang

**Affiliations:** 1 Laboratory of Food Science and Human Health, College of Food Science and Engineering, Ocean University of China, Qingdao 266003, China; 2 Laboratory of Animal Genomics and Improvement, United States Department of Agriculture, Agriculture Research Service (USDA-ARS), Beltsville, MD 20705, USA; 3 Qingdao National Laboratory for Marine Science and Technology, Qingdao 266235, China

**Keywords:** astaxanthin *n*-octanoic acid diester, insulin resistance, inflammation, 16S rRNA, gut microbiota

## Abstract

Astaxanthin *n*-octanoic acid diester (AOD) is a type of astaxanthin connecting medium-chain fatty acids with a more stable structure. In this study, we examined the role of AOD in ameliorating insulin resistance (IR) induced by a high-fat and high-sucrose diet (HFD) as well as its effect on modulating gut microbiota in mice, with free astaxanthin (AST) as a comparison. Four groups of male C57BL/6J mice (6 weeks old; *n* = 10 per group) were fed with a normal control diet (NC), HFD orally administered with AOD, AST (50 mg/kg body weight), or vehicle for 8 weeks. AOD improved glucose tolerance, IR, systematic and intestinal inflammation, and intestinal integrity better than AST. Further, both AOD and AST modulated gut microbiota. A significantly higher abundance of *Bacteroides* and *Coprococcus* was found in AOD than in AST, and the predicted pathway of carbohydrate metabolism was significantly impacted by AOD. Overall, AOD may play a role in alleviating IR and inflammation with the modulating effect on microbiota in HFD-fed mice. Our findings could facilitate the development of AOD as a bioactive nutraceutical and more stable alternative to AST.

## 1. Introduction

Due to people’s preference for a high-fat and high-sucrose diet (HFD), chronic metabolic diseases such as insulin resistance (IR), weight gain, and dyslipidemia are becoming increasingly common [1]. IR refers to the weakening response of tissues to circulating insulin [2], which is the main inducing factor of type 2 diabetes, obesity, cardiovascular and neurodegenerative diseases, and several cancers [3]. In the past 10 years, research has revealed that gut microbiota play an important role in IR and the glucose tolerance of host [4]. Lipopolysaccharide (LPS), the product of gut microbiota, plays important roles in the interaction between gut microbiota and IR [3]. As HFD impairs the tight junction of intestinal epithelial cells and increases intestinal permeability [5], endotoxin from microbiota transfer from the bowel lumen into the bloodstream through the impaired intestinal barrier, thereby inducing obesity and leading to IR [3]. These metabolic disorders are associated with a state of chronic, low-grade systemic inflammation, which is caused by the activation of immune cells such as macrophages and T lymphocytes, as well as the production of proinflammatory cytokines such as tumor necrosis factor (TNF), interleukin 1β (IL-1β), and interferon-γ (IFN-γ) [6,7,8,9]. 

**Figure 1:**
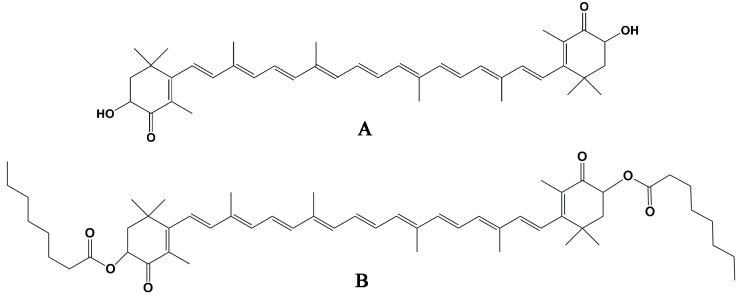
Structures of AST and AOD (according to Fukami et al. [15]). (**A**) AST: free astaxanthin. (**B**) AOD: astaxanthin *n*-octanoic acid diester.

Astaxanthin (AST) is a xanthophyll carotenoid widely distributed in shrimp, crab, algae, and other marine animals and plants in nature [10]. AST bears two ionone rings held together by a series of conjugated carbon–carbon double bonds. AST (Figure 1A) is considered unstable and easily oxidized. Therefore, in nature, it is either conjugated with proteins or esterified with one or two fatty acids (monoester or diester) to be more stable [11]. AST esters are better absorbed in the small intestine than AST, have higher bioavailability, and accumulate more in various tissues [12,13,14]. Fukami et al. synthesized astaxanthin *n*-octanoic acid esters, a type of medium-chain fatty acid esters, through chemical and enzymatic methods [10,15]. The metabolism of astaxanthin *n*-octanoic acid esters in blood and liver has been investigated, and it was found that the oral absorption of astaxanthin *n*-octanoic acid esters was better than that of AST or astaxanthin long-chain fatty acid ester mixture extracted from *Haematococcus* algae [15]. 

The consumption of astaxanthin has brought a variety of benefits to human health, including protection from ultraviolet-light photo-oxidation, preventing or reducing the risk of cancer, as well as enhancement of the immune response in humans and animals [16]. Numerous studies have reported that AST has a significant improvement effect on metabolic syndromes such as IR and glucose tolerance caused by an HFD [17,18,19]. Humans are not capable of synthesizing astaxanthin de novo, and thus they must obtain it through diet [20]. It has been reported that AST has a modulating effect on gut microbiota. Lyu et al. found that AST is related to immunoglobulin A regulation and significantly increased the abundance of *Bifidobacterium* in mice [21]. AST has been found to increase *Akkermansia* and prevent alcoholic fatty liver disease in mice with alcoholic fatty liver disease [22]. However, due to the structural instability of AST, it is easily oxidized, which adversely affects its production and application. The purpose of this study was to find a suitable alternative to astaxanthin that would have higher stability without disrupting its improving role in metabolic syndrome. It has been reported that astaxanthin *n*-octanoic acid diester (AOD, Figure 1B) can be synthesized by enzymatic and chemical methods and is more stable than AST with a higher absorption rate [10,15]. In this study, we conducted animal experiments to evaluate the prevention and ameliorating effects of AOD and AST on HFD-induced IR, and the modulating effects on gut microbiota by 16S rRNA gene high-throughput sequencing.

## 2. Results

### 2.1. AOD Attenuated Glucose Intolerance and IR

**Figure 2:**
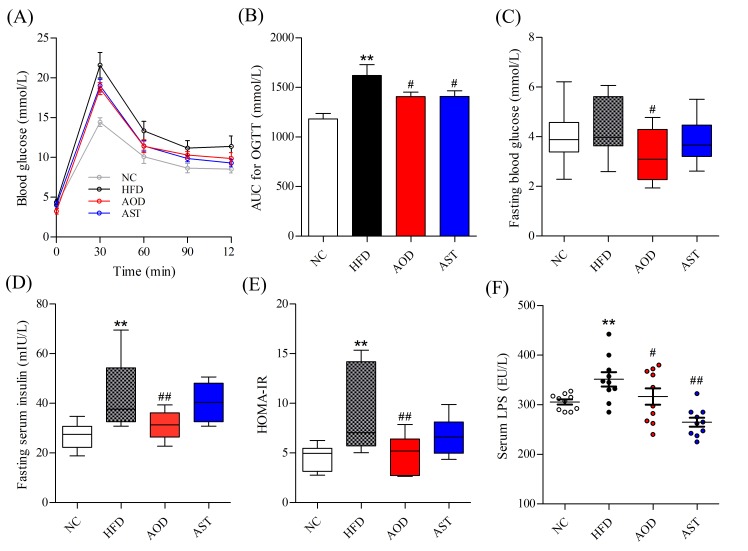
AOD ameliorated glucose intolerance and insulin resistance. (**A**) Blood glucose levels during oral glucose tolerance test (OGTT) at week 8. (**B**) Blood glucose area under the curve (AUC) for OGTT. (**C**) Fasting blood glucose level. (**D**) Fasting serum insulin level. (**E**) homeostasis model assessment of insulin resistance (HOMA-IR). (**F**) Serum lipopolysaccharide (LPS) level. NC: normal control diet; HFD: high-fat and high-sucrose diet; AOD: high-fat and high-sucrose diet supplemented with astaxanthin *n*-octanoic acid diester (50 mg/kg body weight) for 8 weeks; AST: high-fat and high-sucrose diet supplemented with free astaxanthin (50 mg/kg body weight) for 8 weeks. The values are presented as mean ± SEM (standard error of mean), *n* = 10. ** *p* < 0.01, vs. NC, and # *p* < 0.05, ## *p* < 0.01 vs. HFD group.

There were no differences in energy intake and weight gain in AOD and AST groups compared to the HFD group during the study period (Figure S1). However, the administration of AOD and AST significantly improved glucose tolerance compared to the HFD group (Figure 2A). Compared to the mice fed with a normal diet, HFD significantly increased the area under the curve (AUC) in the oral glucose tolerance test (OGTT), whereas mice treated with AOD and AST had a reduced AUC by approximately 13% (*p* < 0.05; Figure 2B). The fasting serum glucose level was also significantly suppressed by AOD (Figure 2C). HFD-feeding significantly increased the fasting serum insulin level by 60.64% compared with the normal control (NC) group (Figure 2D). AOD as a dietary supplement significantly decreased the fasting insulin serum level by 28.09% (*p* < 0.01; Figure 2D). The level of homeostasis model assessment of insulin resistance (HOMA-IR) was found to be almost doubled in the HFD group compared with the NC group (*p* < 0.01), and AOD decreased the HOMA-IR level by 48.20% (*p* < 0.01; Figure 2E). In contrast, AST slightly decreased the fasting glucose level, insulin level, and HOMA-IR, but no significance was observed (*p* > 0.05). Further, HFD-feeding significantly increased serum LPS level compared with the NC group; however, these LPS levels returned to a normal level in AOD and AST groups (*p* < 0.05 and *p* < 0.01, respectively; Figure 2F).

### 2.2. AOD Relieved Intestinal Oxidative Stress and Inflammation 

**Figure 3:**
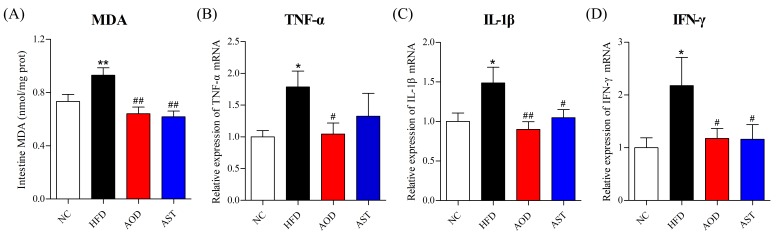
AOD improved the malondialdehyde (MDA) level and the expression levels of pro-inflammatory cytokines in the jejunum. (**A**) MDA level. (**B**–**D**) mRNA expression levels of tumor necrosis factor (TNF)-α, interleukin 1β (IL-1β), and interferon-γ (IFN-γ). NC: normal control diet; HFD: high-fat and high-sucrose diet; AOD: high-fat and high-sucrose diet supplemented with astaxanthin *n*-octanoic acid diester (50 mg/kg body weight) for 8 weeks; AST: high-fat and high-sucrose diet supplemented with free astaxanthin (50 mg/kg body weight) for 8 weeks. The values are presented as mean ± SEM, *n* = 10. * *p* < 0.05, ** *p* < 0.01 vs. NC, and # *p* < 0.05, ## *p* < 0.01 vs. HFD group.

We evaluated the common oxidative stress index—malondialdehyde (MDA), and the expression of mRNA encoding inflammatory cytokines. Our results showed that AOD significantly ameliorated intestinal oxidative stress and inflammation (Figure 3). The content of MDA was significantly decreased by AOD and AST compared to the HFD group (Figure 3A). The expression levels of mRNA encoding inflammatory cytokines, including TNF-α, IL-1β, and IFN-γ, were higher in the HFD group than in the NC group; however, these mRNAs were significantly downregulated in the AOD group, and AST downregulated the expression of IL-1β and IFN-γ when compared to the HFD group (Figure 3B–D).

### 2.3. AOD Upregulated Tight Junction Components

**Figure 4:**
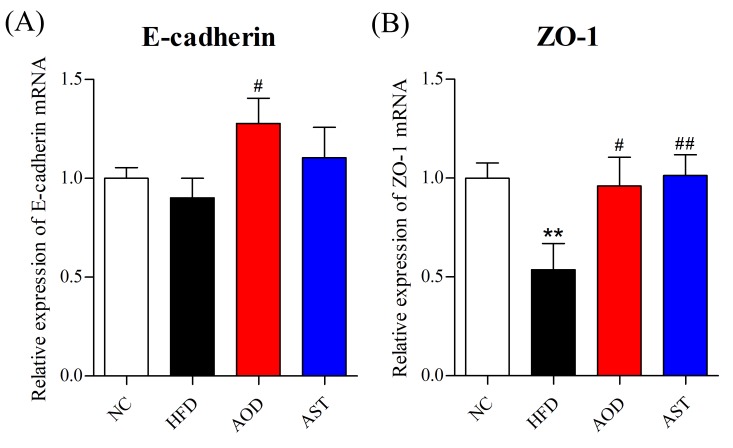
AOD upregulated the expression levels of tight junction components in the jejunum. mRNA expression levels of (**A**) E-cadherin and (**B**) zonula occludens 1 (ZO-1) in the jejunal tissue. NC: normal control diet; HFD: high-fat and high-sucrose diet; AOD: high-fat and high-sucrose diet supplemented with astaxanthin *n*-octanoic acid diester (50 mg/kg body weight) for 8 weeks; AST: high-fat and high-sucrose diet supplemented with free astaxanthin (50 mg/kg body weight) for 8 weeks. The values are presented as mean ± SEM, *n* = 10. ** *p* < 0.01 vs. NC, and # *p* < 0.05, ## *p* < 0.01 vs. HFD group.

We analyzed the expression levels of mRNA encoding adherens junction protein E-cadherin and tight junction components zonula occludens 1 (ZO-1). The expression levels of E-cadherin and ZO-1 mRNAs were increased in the AOD group compared with those in the HFD group (Figure 4), and AST reversed the expression of ZO-1 (Figure 4B) compared to HFD.

### 2.4. HFD Induced a Profound Change in the Gut Microbiota

The mean number of observed operational taxonomic units (OTUs) per sample identified in this study was 632.80 ± 47.61 (mean ± SD; *n* = 40; Table S1). Compared to mice in the NC group, HFD slightly reduced the number of observed OTUs and other microbial α-diversity indices, such as Chao1, Shannon’s, Simpson’s, and phylogenetic diversity (PD) whole tree (*p* > 0.05).

**Figure 5:**
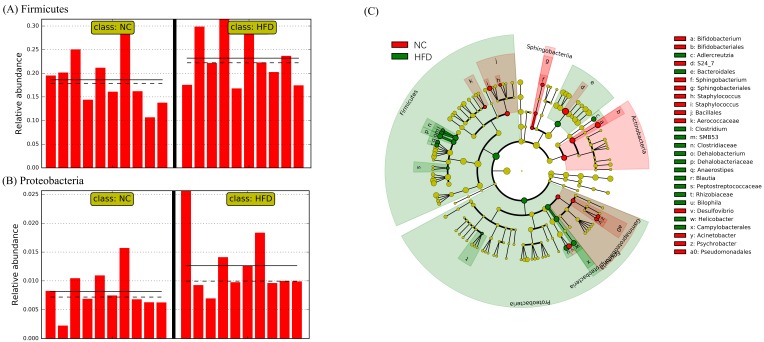
Select microbial taxa displaying significantly different abundance between NC and HFD groups. The two phyla significantly impacted by HFD are depicted in (**A**) Firmicutes and (**B**) Proteobacteria. *x*-axis: individual samples. *y*-axis: relative abundance percentage. Straight line: group mean abundance. Dotted line: median. Taxa meeting a significant LDA threshold value >2.0 and its corresponding taxonomic cladogram are depicted in (**C**). NC: normal control diet; HFD: high-fat and high-sucrose diet.

At the phylum level, the abundance of Firmicutes and Proteobacteria was significantly increased in the HFD group (log_10_ linear discriminant analysis (LDA) score = 4.62 and 4.07, respectively) as compared to that in the NC group (Figure 5A,B). Linear discriminant analysis (LDA) effect size (LEfSe) analysis detected 33 genera that were significantly impacted by HFD (absolute log_10_ LDA score >2.0). Compared with the NC group, the abundance of 16 genera was significantly repressed in the HFD group, whereas the abundance of 17 genera were elevated. Selected genera significantly impacted by HFD are illustrated in Figure 5C. Notably, the abundance of several genera—such as *Bilophila*, *Helicobacter*, *Clostridium*, *Dehalobacterium*, and *SMB53*—were significantly increased by HFD.

Moreover, HFD induced a profound change in the gut microbiota at the species level. The abundance of approximately 16% of the OTUs was significantly altered in the HFD group. Compared to the NC group, HFD significantly repressed the abundance of 53 OTUs, whereas the abundance of 48 OTUs was elevated. Further, the abundance of at least 20 OTUs assigned to the family S24-7 was significantly repressed by HFD.

### 2.5. AOD and AST Modulated Gut Microbiota Under an HFD

**Figure 6:**
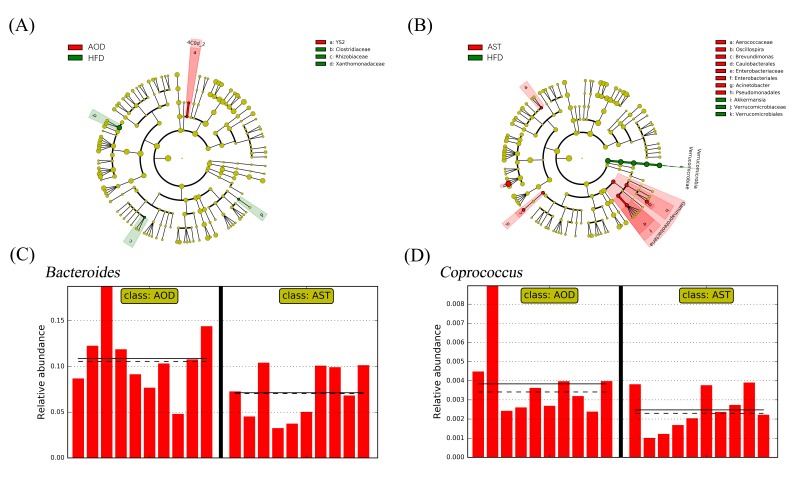
Select taxa with significant difference in abundance of AOD, AST, and HFD samples using linear discriminant analysis (LDA) effect size (LEfSe) analysis. Taxa meeting a significant LDA threshold value of >2.0 and its corresponding taxonomic cladogram are depicted in (**A**) AOD vs. HFD groups (red: AOD-enriched taxa; green: HFD-enriched taxa) and (**B**) AST vs. HFD groups (red: AST-enriched taxa; green: HFD-enriched taxa). (**C**) *Bacteroides* and (**D**) *Coprococcus* are selected microbial genera displaying significant differences in their relative abundance in the gut microbiota between AOD and AST. *x*-axis: individual samples. *y*-axis: relative abundance. Straight line: group mean abundance. Dotted line: median. HFD: high-fat and high-sucrose diet; AOD: high-fat and high-sucrose diet supplemented with astaxanthin *n*-octanoic acid diester (50 mg/kg body weight) for 8 weeks; AST: high-fat and high-sucrose diet supplemented with free astaxanthin (50 mg/kg body weight) for 8 weeks.

The gavage of AOD and AST significantly altered the composition of gut microbiota compared to HFD, with a significant change in the relative abundance of at least 5 and 14 genera, respectively (Figure 6A,B). At the genus level, AOD increased the abundance of two genera belonging to the phylum Cyanobacteria. LEfSe analysis revealed that the genus *Akkermansia* belonging to the phylum of Verrucomicrobia was reduced by AST, whereas the abundance of *Oscillospira*, *Acinetobacter*, and a genus assigned to the family Aerococcaceae were significantly increased. Further, six genera displayed significant differences between the AOD and AST groups. For example, AOD significantly increased the abundance of *Bacteroides* and *Coprococcus* compared to AST (log_10_ LDA score = 4.24 and 2.84, respectively; (Figure 6C,D).

The dietary treatment by AOD and AST slightly increased the richness of the microbial species, but no significance was observed. AOD and AST affected the abundance of 26 and 66 OTUs, respectively, compared to the HFD group. Among them, at least four and six OTUs in the AOD and AST groups, respectively, displayed a reverse change in their abundance compared to the significantly changed OTUs under an HFD (Table 1). Notably, AOD restored two OTUs (GreenGene IDs #389282 and #780650) belonging to the families S24-7 and Clostridiaceae, respectively. Similarly, three OTUs (GreenGene IDs #1788776, #181683, and #4289858) belonging to the order Clostridiales, which were significantly increased in the HFD group compared to NC, were significantly decreased by AST. The relative abundance of at least two OTUs in the AOD and AST groups showed the same change compared to the HFD group. For example, GreenGene ID #166226, assigned to the genus *Oscillospira*, was reduced 242-fold by HFD but increased 70-fold and 150-fold, respectively, by AOD and AST. Similarly, GreenGene ID #351972, belonging to *Dehalobacterium*, was increased from 0.06% to 0.2% by HFD and decreased to 0.05% and 0.06% by AOD and AST, respectively, to a level close to that of the NC group.

### 2.6. Microbial Pathways Impacted by AOD and AST

**Figure 7:**
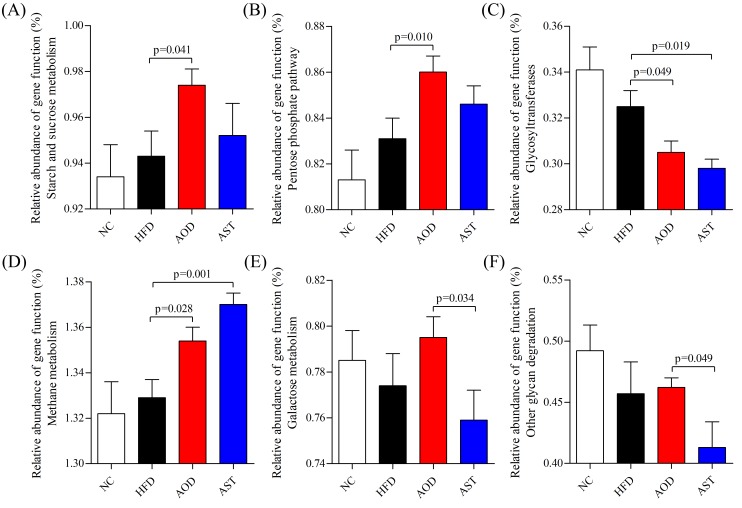
Select biological pathways and functional categories of gut microbiota displaying differences in relative abundance of gene function (%). (**A**) Starch and sucrose metabolism. (**B**) Pentose phosphate pathway. (**C**) Glycosyltransferases. (**D**) Methane metabolism. (**E**) Galactose metabolism. (**F**) Other glycan degradation. NC: normal control diet; HFD: high-fat and high-sucrose diet; AOD: high-fat and high-sucrose diet supplemented with astaxanthin *n*-octanoic acid diester (50 mg/kg body weight) for 8 weeks; AST: high-fat and high-sucrose diet supplemented with free astaxanthin (50 mg/kg body weight) for 8 weeks. The values are presented as mean ± SEM, *n* = 10.

Using the Phylogenetic Investigation of Communities by Reconstruction of Unobserved States (PICRUSt) [23], the 16S rRNA gene sequence data was analyzed to estimate the biological function in the fecal microbial community impacted by AOD and AST. A total of 328 Kyoto Encyclopedia of Genes and Genomes (KEGG) pathways were predicted. AOD and AST were found to have effects on a range of biological functions, particularly on carbohydrate metabolism and glycan biosynthesis and metabolism when compared with HFD. AOD significantly enhanced certain pathways in the carbohydrate metabolism, such as starch and sucrose metabolism (log_10_ LDA score = 2.19) and the pentose phosphate pathway (log_10_ LDA score = 2.13) compared with the HFD group (Figure 7A,B). Specifically, both AOD and AST decreased the abundance of glycosyltransferases (GTs) and increased the abundance of genes involved in methane metabolism compared to the HFD group (Figure 7C,D). Further, it must be noted that the relative abundance of genetic functions related to galactose metabolism and other glycan degradation was significantly higher in the AOD group than in the AST group (log_10_ LDA score = 2.23 and 2.39, respectively; Figure 7E,F).

## 3. Discussion

An HFD causes several health complications such as damaged intestinal barrier, increased translocation of intestinal microbiota, inflammation, and IR [1,24]. AST has been reported to have an effect that causes significant improvement on HFD-induced metabolic syndrome, such as IR and glucose tolerance [17,18,19]. However, the instability of the AST structure and its susceptibility to oxidation have limited its production and application. In this study, we found that AOD has a better improvement effect than AST on IR associated with HFD. Further, the administration of AOD reversed HFD-induced oxidative stress injury and inflammation, as supported by the reduced MDA level and the expression levels of inflammatory cytokines (e.g., TNF-α, IL-1β, and IFN-γ). Similar antidiabetic and anti-inflammatory effects were found in astaxanthin polyethylene glycol succinate conjugates, which is a synthetic hydrophilic astaxanthin with significantly enhanced solubility and stability compared with AST [25]. Further study was conducted around gut microbiota in our research. The effects of AOD on the gut microbiota was studied, and a link between gut microbiota and the alleviating effects on glucose intolerance and inflammation was established.

The gut barrier is the first line of defense between luminal content and host tissue. When the intestinal integrity is impaired, microbiota can come in contact with the intestinal mucosa and stimulate inflammation through the immune system [26,27]. Epithelial junctions are established through E-cadherin-based interactions between adjacent cells and by their association with the cytoskeleton, particularly with the actomyosin machinery and junctional proteins such as ZO-1 [28]. The upregulated E-cadherin and ZO-1 mRNAs indicated an ameliorating effect of AOD on intestinal integrity, which may be one of the reasons for the alleviated effect on inflammation and IR. As a product of gut microbiota, LPS plays an important role in the interaction between gut microbiota and IR [3]. Our data showed that AOD and AST lowered the concentration of LPS to a normal level, thereby suggesting an ameliorating effect on IR through the modulation of gut microbiota. The gut microbiota plays an important role in the development of IR and inflammation [4,29,30]. It has been widely reported that HFD can have a profound impact on the structure and function of the gut microbiota [31,32,33]. In this study, HFD increased the abundance of Firmicutes (LDA score > 2.0), which was in agreement with previous studies [34,35]. The elevated Proteobacteria level was observed in people with metabolic disorders, inflammation, or cancer [36]. At the genus level, we found that *Bilophila*, *Helicobacter*, *Clostridium*, *Dehalobacterium*, and *SMB53* were significantly increased after treatment with HFD. The genus *Helicobacter*, a potential producer of intracellular reactive oxygen species and MDA, induces gastric inflammation and gastrointestinal infection [37,38]. Moreover, an elevated abundance of *Clostridium* and *SMB53* due to an HFD was in agreement with a previous report [39].

In the present study, the results of LEfSe analysis indicated that imbalances in the microbiota occurred in the HFD group, whereas AOD and AST treatment alleviated these imbalances. AOD increased the abundance of two genera belonging to the phylum Cyanobacteria, which is consistent with the effect of orlistat compared with that of the HFD-associated gut community [40]. AST significantly decreased the relative abundance of phylum Verrucomicrobia and its genus *Akkermansia* compared to the HFD group. An increased abundance of *Akkermansia* was reported in rats on an HFD [41,42,43]. It has also been reported that Verrucomicrobia exhibits proinflammatory properties, and their abundance was increased in dextran sodium sulfate-induced colitis mice [44,45]. In the AST group, we observed an increased abundance of *Acinetobacter* and another genus assigned to the family Aerococcaceae, which were related to the upregulation of antibacterial peptide expression and the improvement of inflammatory response, metabolism, and intestinal barrier function in diet-induced obese mice [46,47]. In addition, there were a few differences in the effects of AOD and AST on gut microbiota. For example, the abundance of *Bacteroides* and *Coprococcus* was significantly increased in the AOD group as compared to that in the AST group. *Bacteroides* are closely related to carbohydrate metabolism. It has been reported that some *Bacteroides* species have an extensive starch utilization system, with multiple genes involved in starch binding and utilization, thereby producing various enzymes for carbohydrate metabolism [48]. Further, *Coprococcus* exhibited activity in alleviating oxidative damage [49], providing anti-inflammatory effects, and improving gut barrier function [50] in certain reports, and their abundance was decreased in mice with acute colitis [49]. 

At the species level, AOD enhanced an OTU assigned to the family S24-7, which was significantly repressed in the HFD group. S24-7 is a kind of carbohydrate-fermenting bacteria that produces SCFAs [51]. AST reversed the abundance of three OTUs belonging to the order Clostridiales, which were significantly increased by HFD. The order Clostridiales, a kind of branched-chain amino acid-producing bacteria, has a positive effect on glucose intolerance in HFD-fed mice [52]. Both AOD and AST increased the abundance of an OTU (GreenGene ID #166226) assigned to the genus *Oscillospira* compared with HFD. *Oscillospira* appears to be negatively correlated with inflammatory bowel disease [53] and steatohepatitis [54]. In addition, an OTU (GreenGene ID #351972) assigned to *Dehalobacterium*, which is positively related with the intake of carbohydrate [55], was also repressed by both AOD and AST compared to HFD.

The PICRUSt algorithm enables us to infer functional categories impacted by AOD and AST in the fecal microbiota. Our findings showed that both AOD and AST decreased the abundance of glycosyltransferases and increased the abundance of methane metabolism. GTs are powerful tools for the synthesis of a diverse range of saccharides and glycoconjugates [56]. The decrease in GTs may help to reduce the synthesis of carbohydrate. Methane production possibly plays some roles in metabolic control. It has been reported that people who produce more methane than normal showed a worse glycemic control [57]. Further, the increased abundance of genes in methane metabolism may reduce the production of methane and, hence, help to ameliorate carbohydrate metabolism disorder induced by an HFD. 

Interestingly, the gut microbiota of AOD appeared to utilize more carbohydrate metabolism than AST. AOD significantly enhanced the abundance of starch and sucrose metabolism and pentose phosphate pathway, which helped boost the catabolism of the carbohydrate intake, lower blood glucose levels, and relieve IR. It must be noted that the abundance of galactose metabolism and other glycan degradation pathways was significantly higher in the AOD group than that in the AST group. As a key source of energy, the ingestion of relatively large amounts of galactose (35–50 g) resulted in an increase of less than 1 mmol/L in peripheral glucose concentration, whereas the insulin concentration increased approximately fourfold [58,59], which indicated a significant effect of galactose on IR. Many kinds of glycans have been reported to be beneficial for improving glucose tolerance and IR [60,61]. A higher abundance of other glycan degradation may be beneficial for relieving IR. The differences in predicted pathways affected by microbiota may be one of the reasons why AOD had a better improving effect on IR than AST. The PICRUSt algorithm provides a rapid snapshot on how dietary supplements shape the biological functions of gut microbiota. However, this approach is mainly based on human database. Studies including transcriptomics and metabolomics are needed for further validation. In addition, the improvement of astaxanthin on insulin resistance is also related to other mechanisms, such as adipokine levels. It has been reported that astaxanthin can diminish the increase of pro-inflammatory adipokines monocyte chemoattractant protein-1 and vascular endothelial growth factor and increase serum adiponectin level [62,63,64]. More research needs to be done around these aspects.

Overall, our data suggest that AOD possesses the potential to prevent and ameliorate IR associated with an HFD. Our findings emphasize the importance of AOD as a potential bioactive compound and suitable substitute for astaxanthin in modulating host glucose levels and restoring the gut microbiota associated with an HFD.

## 4. Materials and Methods 

### 4.1. Preparation of AOD

AST (HPLC purity >97%) was purchased from Dr. Ehrenstorfer (Augsbury, Germany). The synthesis procedure of AOD was as follows [15]. Under the protection of nitrogen, AST and *n*-octanoic acid reacted under the catalysis of 4-dimethylaminopyridine and 1-(3-dimethylaminopropyl)-3-ethylcarbodiimide hydrochloride. After the reaction for 12 h at room temperature (25 °C), dichloromethane was added to the solution above. The mixture was then washed successively by 1 M hydrochloric acid, saturated sodium bicarbonate, and saturated sodium chloride. The solvent was dried with anhydrous sodium sulfate, and the organic phase was removed under reduced pressure to obtain the AOD (HPLC purity >90%), which was confirmed by high-performance liquid chromatography-diode array detector (HPLC-DAD) according to the previously published study [65].

### 4.2. Animal Maintenance and Treatment

All experiment protocols were conducted according to the Guide for the Care and Use of Laboratory Animals of the National Institutes of Health and were approved by the Ethical Committee of Animal Experiments of Ocean University of China (approved protocol no: SPXY2017050401; approval date: 1 May 2017). Animal welfare was assessed according to Laboratory Animal—Guideline for Ethical Review of Animal Welfare (GB/T 35892-2018). Forty Male C57BL/6J mice (6 weeks old, 20–22 g) were obtained from Vital River Laboratory Animal Technology Co. Ltd. (Beijing, China). The mice were individually housed with a 12 h light/dark cycle at 24 ± 2 °C and 65% ± 15% humidity throughout the experiment. The mice were given free access to food and water.

Diets were prepared according to the feed formula provided by Research Diets (New Brunswick, NJ; Table S2). After an adaption period of 1 week, the mice were randomly divided into 4 groups with 10 mice in each group: NC diet (D12450J; 10% calories in fat, 7% calories in sugar), HFD diet (D12451; 45% calories in fat, 17% calories in sugar), HFD with AOD, or AST at a daily dose of 50 mg/kg body weight (AST equivalents). The mice were given corn oil (control) or AOD or AST dissolved in corn oil by oral gavage for 8 weeks.

Body weight data of the mice were recorded weekly during the experiment. At the end of the experiment, the mice were anesthetized and sacrificed by cervical dislocation after overnight fasting. Blood samples were collected. Following sacrifice, the intestine samples were harvested and immediately frozen in liquid nitrogen. Feces were collected from the colon for subsequent 16S rRNA analysis. All samples were stored at −80 °C for further analysis. 

### 4.3. Detection of Serum and Intestinal Parameters

An OGTT was performed after mice were maintained for 8 weeks. After fasting for 12 h, mice were orally administrated 2 g/kg body weight of glucose solution, and blood samples were collected from the tail vein at 0, 30, 60, 90, and 120 min after oral gavage of glucose. Serum glucose level was measured using a commercial kit (Biosino, Beijing, China) following manufacturer’s protocol. The AUC of OGTT was calculated. Serum insulin and LPS levels were determined by ELISA kit (Cusabio technology, Wuhan, China). The Equation (1) was proposed for calculating HOMA-IR:

*HOMA-IR = (fasting serum glucose level × fasting serum insulin level)/*22.5 (1)

In addition, the intestinal MDA level was assayed using a testing kit purchased from Nanjing Jiancheng Bioengineering Institute (Nanjing, China).

### 4.4. Real-Time qPCR

Total RNA from intestinal samples was extracted as previously described [66]. The concentration of each extracted RNA sample was determined using a Nano Drop 2000 Spectrophotometer (Thermo, USA), and the integrity of the RNA was checked using denatured RNA electrophoresis. Real-time qPCR was performed according to the method of Zhang et al. [67]. The primers were synthesized by Shanghai Sangon Gene Company (Qingdao, China) and the sequences are listed in Table S3. The results were expressed as relative mRNA expression compared to the control mice.

### 4.5. Fecal Total DNA Extraction

The fecal samples collected from the colon lumen at necropsy were used to extract microbial genomic DNA with a QIAamp fast DNA stool mini kit (Qiagen, Hilden, Germany) with some modifications. An 8-min incubation at 95 °C was used to replace the 70 °C lysis recommended in the standard protocol. A Nanodrop 2000 (Thermo, USA) was applied to quantify the concentration and evaluate the purity of DNA.

### 4.6. Sequencing and Bioinformatics Analysis

The hypervariable V3-V4 regions of the 16S rRNA gene were sequenced as previously described [68]. Briefly, 40 ng of total DNA was mixed with 0.7 μL of 10 μM PAGE-purified Illumina platform-compatible adaptor oligos that contain features such as sequencing primers, sample-specific barcodes, and 16S PCR primers (forward primer, 341/357F: NNNNCCTACGGGNGGCWGCAG; reverse primer, 805/785R: GACTACHVGGGTATCTAATCC). The amplification reaction was performed by Phusion high-fidelity PCR Master Mix (New England Biolabs, Ipswich, MA, USA). A BioAnalyzer 7500 DNA chip and a QuantiFluor fluorometer were used to quantify DNA. According to their respective sample-specific barcodes, amplicons were pooled at the same molar ratios. The pool was purified by Agencourt XP beads (Beckman Coulter Genomics, Danvers, MA, USA), and a BioAnalyzer 7500 DNA chip kit (Agilent) was used to quantify the concentration of the final library pool. Approximately 25% of whole-genome shotgun libraries prepared using an Illumina TruSeq DNA sample prep kit with a compatible adaptor barcode was added to the purified amplicon pool. The library pool was sequenced using an Illumina MiSeq Reagent Kit on an Illumina sequencer, as previously described [69]. 

Trimmomatic (v 0.36) was used for removing the four maximally degenerate bases (“NNNN”) in the forward sequence. The trimmed sequences were analyzed by QIIME1 pipeline (v1.9.1) [70]. A “closed-reference” protocol in the pipeline was used for OTUs picking. Sequences with ≥97% similarity were assigned to the same OTU. Taxonomy assignment was performed according to the GreenGene database (v13.8). The QIIME1 and Primer6 programs were used to conduct Alpha(α)-diversity analysis. Further, the LEfSe algorithm [71] was used to identify taxa and KEGG gene families and pathways that display significant differences between two biological conditions. Further, on the basis of the OTU table generated using the closed-reference protocol in QIIME1, the PICRUSt algorithm (v1.1.1) [23]—a software package designed to predict metagenome functional contents from marker gene surveys—was used with default parameters to predict gene contents and metagenomic functional information. Briefly, the OTU table was first normalized by dividing each OTU by the known/predicted 16S copy number by using the PICRUSt workflow: normalize_by_copy_number.py. The gene contents or the abundance of KEGG Orthology were predicted from the normalized OTU table using the following workflow: predict_metagenomes.py. The predicted metagenome function was further analyzed by collapsing thousands of KEGG orthologs into higher functional categories (pathways) (categorize_by_function.py). In addition, specific OTUs contributing to a given function or pathway were identified by using the following work flow: metagenome_contributions.py, as described previously [68].

### 4.7. Statistical Analysis

Data in the tables and figures are expressed as mean ± SEM (standard error of mean). Statistical analysis was conducted through one-way analysis of variance (ANOVA) using IBM SPSS v18.0. A *p*-value < 0.05 was considered statistically significant and a *p*-value <0.01 was considered extremely significant. In addition, the LEfSe method [71] was used to test the significant differences in microbiome features or pathways between two groups, and the cutoff value is set to be absolute log_10_ LDA score >2.0. All the graphs were made using the GraphPad Prism 5.01. 

## Figures and Tables

**Table 1:** Operational taxonomic units (OTUs) significantly impacted by AOD and AST in the gut microbiome in mice.

OTU_ID (GreenGene)	Annotation	NC	HFD	AOD	AST	Significant
389282	(*S24*-*7*)	0.0977 ± 0.0676	0.0089 ± 0.0021	0.1460 ± 0.1110	0.0380 ± 0.0200	ab
780650	(*Clostridiaceae*)	0.2871 ± 0.0918	1.2600 ± 0.3257	0.3875 ± 0.1396	0.7526 ± 0.1670	ab
166226	*Oscillospira sp,*	0.2080 ± 0.0738	0.0009 ± 0.0006	0.0599 ± 0.0271	0.1293 ± 0.0510	abc
351972	*Dehalobacterium sp,*	0.0613 ± 0.0324	0.2008 ± 0.0421	0.0457 ± 0.0265	0.0633 ± 0.0229	abc
1788776	(*Clostridiaceae*)	0.0000 ± 0.0000	0.0005 ± 0.0002	0.0000 ± 0.0000	0.0000 ± 0.0000	ac
181683	(*Ruminococcus*) *gnavus*	0.0000 ± 0.0000	0.0003 ± 0.0001	0.0002 ± 0.0001	0.0000 ± 0.0000	ac
338258	(*S24*-*7*)	0.8171 ± 0.1085	0.0978 ± 0.0886	0.1681 ± 0.0853	0.4071 ± 0.1099	ac
4289858	(*Clostridiaceae*)	0.0000 ± 0.0000	0.0003 ± 0.0001	0.0000 ± 0.0000	0.0000 ± 0.0000	ac

NC: normal control diet; HFD: high-fat and high-sucrose diet; AOD: high-fat and high-sucrose diet supplemented with astaxanthin *n*-octanoic acid diester (50 mg/kg body weight) for 8 weeks; AST: high-fat and high-sucrose diet supplemented with free astaxanthin (50 mg/kg body weight) for 8 weeks. The numbers denote the relative abundance. Data are mean ± SEM (*n* = 10). Significant means that the abundance of the OTU that was changed at a cutoff value of the absolute log_10_ LDA scores > 2.0 between the two contrast groups using linear discriminant analysis (LDA) effect size (LEfSe) algorithm (a = NC vs. HFD; b = AOD vs. HFD; c = AST vs. HFD).

## Supplementary Materials

Supplementary materials can be found at https://www.mdpi.com/1422-0067/21/6/2149/s1. Figure S1: Effect of astaxanthin on body weight and food intake of mice. Table S1: The OTUs per sample identified in this stud. Table S2: Composition of rodent diets. Table S3: Primer sequences for RT-PCR amplification.

## Abbreviations

AOD	Astaxanthin *n*-octanoic acid diester
AST	Free astaxanthin
AUC	Area under the curve
GTs	Glycosyltransferases
HFD	High-fat and high-sucrose diet
HPLC-DAD	High-performance liquid chromatography-diode array detector
HOMA-IR	Homeostasis model assessment of insulin resistance
IFN-γ	Interferon-γ
IL-1β	Interleukin 1β
IR	Insulin resistance
KEGG	Kyoto Encyclopedia of Genes and Genomes
LDA	The linear discriminant analysis
LEfSe	Linear discriminant analysis effect size
LPS	Lipopolysaccharide
NC	Normal control diet
OGTT	Oral glucose tolerance test
OTUs	Operational taxonomic units
PD	Phylogenetic diversity
PICRUSt	Phylogenetic Investigation of Communities by Reconstruction of Unobserved States
SCFAs	Short chain fatty acids
TNF	Tumor necrosis factor
ZO-1	Zonula occludens 1
