# Supplementary material for: Astaxanthin n-Octanoic Acid Diester Ameliorates Insulin Resistance and Modulates Gut Microbiota in High-Fat and High-Sucrose Diet-Fed Mice
Source: Int J Mol Sci. 2020 Mar 20;21(6):2149. doi: 10.3390/ijms21062149 (PMC7139465; doi:10.3390/ijms21062149)
Supplement: Supplementary file 1 [file ijms-21-02149-s001.zip › Supplementary Files/Figure S1.docx]

**
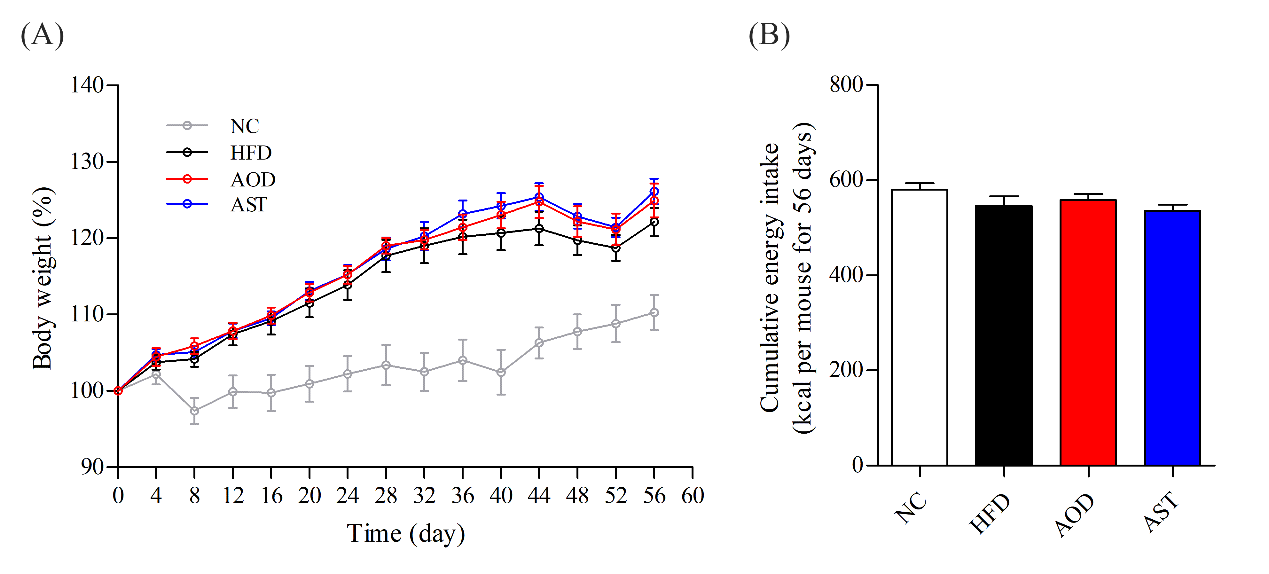
**

**Fig. S1.** Effect of astaxanthin on body weight and food intake of mice. (**A**) Body weight changes during the 8 weeks. (**B**) Cumulative energy intake for 8 weeks. NC: normal control diet; HFD: high-fat and high-sucrose diet; AOD: high-fat and high-sucrose diet supplemented with astaxanthin *n*-octanoic acid diester (50 mg/kg body weight) for 8 weeks; AST: high-fat and high-sucrose diet supplemented with free astaxanthin (50 mg/kg body weight) for 8 weeks. The values are presented as mean ± SEM (standard error of mean), *n* = 10.
