# Supplementary material for: Astaxanthin n-Octanoic Acid Diester Ameliorates Insulin Resistance and Modulates Gut Microbiota in High-Fat and High-Sucrose Diet-Fed Mice
Source: Int J Mol Sci. 2020 Mar 20;21(6):2149. doi: 10.3390/ijms21062149 (PMC7139465; doi:10.3390/ijms21062149)
Supplement: Supplementary file 1 [file ijms-21-02149-s001.zip › Supplementary Files/Table S2.docx]

**Table S2.** Composition of rodent diets.

| **Diet** | **NC** | | **HFD** | |
| --- | --- | --- | --- | --- |
| *Product #* | *D12450J* | | *D12451* | |
| % | *gm* | *kcal* | *gm* | *kcal* |
| Protein | 19.2 | 20 | 24 | 20 |
| Carbohydrate | 67.3 | 70 | 41 | 35 |
| Fat | 4.3 | 10 | 24 | 45 |
| Total |  | 100 |  | 100 |
| kcal/gm | 3.85 |  | 4.73 |  |
| *Ingredient* | *gm* | *kcal* | *gm* | *kcal* |
| Casein | 200 | 800 | 200 | 800 |
| L-Cysteine | 3 | 12 | 3 | 12 |
| Corn Starch | 506.2 | 2024.8 | 72.8 | 291 |
| Maltodextrin 10 | 125 | 500 | 100 | 400 |
| Sucrose | 68.8 | 275.2 | 172.8 | 691 |
| Cellulose, BW200 | 50 | 0 | 50 | 0 |
| Soybean oil | 25 | 225 | 25 | 225 |
| Lard | 20 | 180 | 177.5 | 1598 |
| Mineral Mix S10026 | 10 | 0 | 10 | 0 |
| Dicalcium Phosphate | 13 | 0 | 13 | 0 |
| Calcium Carbonate | 5.5 | 0 | 5.5 | 0 |
| Potassium Citrate, 1 H_2_O | 16.5 | 0 | 16.5 | 0 |
| Vitamin Mix, V10001 | 10 | 40 | 10 | 40 |
| Choline Bitartrate | 2 | 0 | 2 | 0 |
| **Total** | **1055** | **4057** | **858.1** | **4057** |

NC, normal control diet; HFD, high-fat and high-sucrose diet.
