# Supplementary material for: Astaxanthin n-Octanoic Acid Diester Ameliorates Insulin Resistance and Modulates Gut Microbiota in High-Fat and High-Sucrose Diet-Fed Mice
Source: Int J Mol Sci. 2020 Mar 20;21(6):2149. doi: 10.3390/ijms21062149 (PMC7139465; doi:10.3390/ijms21062149)
Supplement: Supplementary file 1 [file ijms-21-02149-s001.zip › Supplementary Files/Table S3.docx]

**Table S3.** Primer sequences for RT-PCR amplification.

| **Gene** | **Forward** | **Reverse** |
| --- | --- | --- |
| TNF-α | CTGTAGCCCACGTCGTAGC | TTGAGATCCATGCCGTTG |
| IL-1β | ACCTGCTGGTGTGTGACGTT | TCGTTGCTTGGTTCTCCTTG |
| IFN-γ | TACACACTGCATCTTGGCTTTG | CTTCCACATCTATGCCACTTGAG |
| E-cadherin | CAAGGACAGCCTTCTTTTCG | TGGACTTCAGCGTCACTTTG |
| ZO-1 | TACCTCTTGAGCCTTGAACTT | CGTGCTGATGTGCCATAATA |
